# In-Hospital Mortality Risk in Post-Percutaneous Coronary Interventions Cancer Patients: A Nationwide Analysis of 1.1 Million Heart Disease Patients

**DOI:** 10.7759/cureus.9071

**Published:** 2020-07-08

**Authors:** Anusha Gaddam, Temitope Ajibawo, Virendrasinh Ravat, Timiiye Yomi, Rikinkumar S Patel

**Affiliations:** 1 Internal Medicine, Chalmeda Anand Rao Institute of Medical Sciences, Karimnagar, IND; 2 Internal Medicine, Brookdale University Hospital Medical Center, New York City, USA; 3 Internal Medicine, Krishna Institute of Medical Sciences, Karad, IND; 4 Medicine, University of Benin School of Medicine, Benin City, NGA; 5 Psychiatry, Griffin Memorial Hospital, Norman, USA

**Keywords:** cancer, primary pci, primary percutaneous intervention, mortality

## Abstract

Objectives

The primary goal of this inpatient study is to assess the risk of in-hospital mortality due to cancer and chronic comorbidities in post-percutaneous coronary intervention (PCI) patients.

Methods

We conducted a retrospective cross-sectional study, including 1,131,415 adult patients (age +18 years) by using the Nationwide Inpatient Sample (NIS) from 2012 to 2014. These patients underwent PCI, and they were further sub-grouped by the co-diagnosis of cancer. Logistic regression analysis was used to evaluate the risk of association between comorbid cancer and in-hospital mortality in post-PCI inpatients.

Results

Most PCI inpatients with cancer were older adults (mean age 70.6 years), males (71.8%), and white (80.6%). Post-PCI mortality risk was 1.28 times higher in females (95% CI 1.235 - 1.335) as compared to males. Coagulopathy and anemias significantly increased the risk of post-PCI mortality by three times (95% CI 2.837 - 3.250) and 1.6 times (95% CI 1.534 - 1.692), respectively. Comorbid cancer was associated with an increased risk of in-hospital mortality in post-PCI patients by 1.9 times (95% CI 1.686 - 2.086) after controlling for demographic confounders and chronic comorbidities.

Conclusion

Our analysis showed that cancer is an independent risk factor for in-hospital mortality after PCI. This association calls for an integrated care model in the management of a complex patient population with cancer and other comorbidities requiring more vigilance and aggressive management.

## Introduction

Percutaneous coronary intervention (PCI) is one of the most commonly performed interventional procedures with substantial geographic variation in the United States (US) [1]. After the Clinical Outcomes Utilizing Revascularization and Aggressive Drug Evaluations (COURAGE) study focused on the criteria for coronary artery revascularization, the utilization of PCI for stable coronary artery disease (CAD) has reduced significantly. This reduction was mainly attributed to the improvement in optimal medical therapy, lifestyle modification, and increasing use of drug-eluting stents leading to a decline in repeat revascularization [2]. PCI is indicated in the treatment of acute coronary syndrome (ACS) and medically unresponsive chronic stable CAD [3].

The leading cause of death within 30 days of PCI included undifferentiated causes and a small proportion of deaths related to cardiovascular causes. Amongst all cardiovascular-related deaths, a lower proportion was attributed to PCI [4]. Irrespective of PCI indication and extent of CAD, the evaluation of long-term mortality in all age groups also exhibited a significant surge in non-cardiac causes of death. The presence of comorbidities, primarily cancer and chronic diseases, including pulmonary and neurologic conditions, multiorgan failure, and renal failure, contributed to an increase in noncardiac mortality [5].

Cardiovascular disease and cancer are the most common causes of death in the world as per the data of the global burden of disease [6]. The presence of shared risk factors between cancer and CAD, along with common pathogenesis of inflammation and oxidative stress, led to an increase in the number of cancer patients who develop CAD [7]. The prevalent cancers in 2016 were breast, lung and bronchus, prostate, colon, and rectum cancer [8]. PCI is the preferred revascularization approach for most cancer patients with CAD, mainly if their malignancy is aggressive or widespread [9]. Furthermore, a study of cancer patients with ACS indicated a significantly lower in-hospital mortality rate with PCI than that of patients receiving conservative medical treatment [10]. However, the clinical outcomes post-PCI showed a higher risk of cardiac morbidity and death in patients with cancer when compared to patients without cancer. The risk of in-hospital mortality and one-year cardiac mortality, target lesion revascularization, and significant bleeding along with all-cause mortality is also increased in cancer patients when compared to patients without cancer [11]. Hypercoagulability and bleeding risk associated with cancer tend to complicate the treatment of CAD in acute and chronic settings [12]. The prognostic impact of cancer is further specific for the type of cancer, presence of metastasis, and whether the diagnosis is historical or current [13].

In our study, we aim to assess the differences in demographics and chronic comorbidities seen in PCI inpatients by the presence of comorbid cancer. Next is to evaluate the risk of in-hospital mortality due to cancer and chronic comorbidities in post-PCI patients.

## Materials and methods

Data source

We used the Nationwide Inpatient Sample (NIS) from 2012 to 2014 in a retrospective cross-sectional study. The NIS is an administrative database that includes patient health information from about 4,400 non-federal, community-based hospitals across 44 states in the US. Diagnostic and procedural information in the NIS is detected using the International Classification of Diseases, ninth edition (ICD-9) codes. Patient health information and identity were protected, and so using the de-identified NIS database does not require approval from the institutional review board [14].

Inclusion criteria and outcome variables

We included 1,131,415 adult patients (age +18 years) with a primary procedure of PCI using ICD-9 codes 36.06 (non-drug-eluting coronary artery stents) or 36.07 (drug-eluting coronary artery stents). This sample was further sub-grouped based on the co-diagnosis of cancer.

Demographic variables studied included age, sex (male and female), and race (white, black, Hispanic, and others). Comorbid diagnoses of deficiency anemias, coagulopathy, diabetes, hypertension, obesity, renal failure, and coagulopathy were identified using ICD-9 codes. We measured the in-hospital mortality between cancer and non-cancer cohorts, and in the NIS, the in-hospital mortality is reported as all-cause [15].

Statistical analysis

We used descriptive statistics and Pearson's chi-square test to discern the demographic and comorbidities differences in PCI inpatients by comorbid cancer. Logistic regression analysis was used to evaluate the demographics, comorbidities, and co-diagnoses of cancer that may increase the risk of association with in-hospital mortality. A P-value of less than 0.01 was considered statistically significant in all analyses that were done in the Statistical Package for the Social Sciences (SPSS) version 26 (IBM Corp., Armonk, NY).

## Results

We analyzed a total sample of 1,131,415 inpatients managed primarily by PCI, with 1.27% having the co-diagnosis of cancer. The majority of the PCI inpatients with cancer were older than non-cancer patients (70.6y vs. 64.3y, P <0.001). A higher proportion of cancer inpatients were male (71.8%) and white (80.6%). Hypertension was the most prevalent comorbidity in both cohorts, with a statistically non-significant difference (P = 0.986). Comorbid deficiency anemias (22.2% vs. 9.9%) and coagulopathy (6.8% vs. 2.4%) were seen in a significantly higher proportion of cancer inpatients (P <0.001). In contrast, diabetes and obesity were seen in a lower proportion of cancer inpatients, as shown in Table 1.

**Table 1:** Demographics and in-hospital mortality by comorbid cancer in percutaneous coronary interventions inpatients SD: standard deviation

Variable	Cancer (-)	Cancer (+)	Total	P-value
Total inpatients	1117065	14350	1131415	-
Mean age, years (SD)	64.3 (12.46)	70.6 (10.17)	-	<0.001
Sex, in %				
Male	67.4	71.8	67.4	<0.001
Female	32.6	28.2	32.6	
Race, in %				
White	76.6	80.6	76.7	<0.001
Black	8.8	8.5	8.8	
Hispanic	7.6	5.1	7.5	
Asian	2.3	2.2	2.3	
Native American	0.6	0.5	0.6	
Other	4.0	3.1	4.0	
Comorbidities, in %				
Deficiency anemias	9.9	22.2	10.1	<0.001
Coagulopathy	2.4	6.8	2.5	<0.001
Diabetes	31.7	28.2	31.6	<0.001
Hypertension	75.7	74.7	74.7	0.986
Obesity	16.9	9.5	16.8	<0.001
In-hospital mortality, in %	1.0	2.8	1.1	<0.001

Females had a 28% higher risk (95% CI 1.235 - 1.335) of post-PCI mortality as compared to males. There was statistically no significant association between race and mortality (P = 0.756). Coagulopathy and deficiency anemias increased the risk of post-PCI mortality by three times (95% CI 2.837 - 3.250) and 1.6 times (95% CI 1.534 - 1.692), respectively. There was a statistically significant difference in post-PCI mortality between cancer (2.8%) and non-cancer inpatients (1%, P <0.001). Cancer significantly increases the risk of post-PCI mortality by 1.9 times (95% CI 1.686 - 2.086) as compared to the non-cancer cohort after controlling for demographic and other comorbidities, as shown in Table 2.

**Table 2:** In-hospital mortality risk in percutaneous coronary interventions inpatients

Variable	Logistic regression model			
	Odds ratio	95% confidence interval		P-value
		Lower	Upper	
Age	1.05	1.046	1.049	<0.001
Female	1.28	1.235	1.335	<0.001
Race	1.00	0.986	1.019	0.756
Cancer				
No	Reference			
Yes	1.88	1.686	2.086	<0.001
Comorbidities				
No comorbidity	Reference			
Deficiency anemias	1.61	1.534	1.692	<0.001
Coagulopathy	3.04	2.837	3.250	<0.001
Diabetes	1.02	0.976	1.061	0.417
Hypertension	0.51	0.490	0.531	<0.001
Obesity	0.76	0.718	0.813	<0.001

## Discussion

Cancer patients constitute a growing and high-risk patient population admitted for PCI. According to our study, the majority of cancer patients were older, male (71.8%), and white (80.6%). Worldwide, the majority of cancers occur in the older population, with about 70% prevalent in individuals aged above 50 years [16]. The lifetime probability of being diagnosed with an invasive cancer is to some extent higher for men (39.3%) than for women (37.7%) [17]. The sex disparity in cancer exists due to differences in environmental exposures, endogenous hormones, immune function, and response, along with complex interactions between these influences [18]. There is also a considerable variation in the occurrence of cancer and the outcomes in different racial and ethnic groups. Our study showed a higher cancer incidence among whites compared to other races.

On the contrary, a study using Surveillance, Epidemiology, and End Results (SEER) data from 1973 to 2013, with a sample size of 997,454, showed a higher cancer incidence and mortality among blacks as compared to whites [19]. Blacks were usually younger at diagnosis, had more frequent late-stage diagnoses, and received less aggressive treatments. However, potential confounding factors, such as access to care, physical functioning, and comorbidities, and cancer incidence in other races and ethnic groups were not considered limiting the results observed [20].

The improved survival of cancer patients increased their probability of developing CAD, often requiring PCI. Currently, cancer patients constitute a higher proportion of the PCI population: one in every 13 patients [11]. More than 50% of cancer patients in the US tend to develop anemia during the course of illness. Overexpression of inflammatory cytokines, leading to a shortened survival of red blood cells, suppression of erythroid progenitor cells, impaired iron utilization, and inadequate erythropoietin production, majorly contribute to anemia in cancer. Other factors, including tumor-associated bleeding, hemolysis, chemotherapy, and nutritional deficiencies, lead to a further increase in both ischemic and hemorrhagic risk [21]. The pathogenesis of blood coagulation activation in cancer is complex and multifactorial. Cancer cells influence the expression of inflammatory cytokines and tissue factor, hemostatic proteins, proangiogenic factors, procoagulant microparticles, and adhesion molecules, leading to hypercoagulability. There exists a cyclic relationship, as cancer cells promote thrombosis and clotting proteins, which support cancer growth [22].

Post-PCI, females tend to have a two times higher risk of in-hospital all-cause mortality and 1.5 times the risk of one-year all-cause death as compared to men. These findings are similar to results in our study, which showed that female post-PCI inpatients had 1.3 times higher risk of in-hospital mortality. These observed differences may be attributed to a poorer baseline cardiovascular risk profile, older age at presentation, and a higher prevalence of diabetes, hypertension, and dyslipidemia among women than men [23]. Current evidence of the impact of race/ethnicity in post-PCI mortality is limited. A study of 769,502 multi-vessel PCI-related hospitalizations found racial disparities with non-whites having lower resource utilization and a greater increase in all-cause mortality post-PCI than whites [24]. Another study observed that there was no impact of race/ethnicity on the incidence of acute and one-year post-PCI adverse outcomes despite differences in patient demographics, clinical presentation, angiographic characteristics, and treatment strategies [25]. In our study, we found that race has a non-significant association with in-hospital mortality in post-PCI inpatients.

Comorbid anemia in cancer patients undergoing PCI is associated with a higher risk of major adverse cardiac events and increased long-term mortality. The pathogenesis of anemia, particularly in patients with coronary artery stenosis, involves decreased oxygen delivery to the myocardium leading to ischemia through mismatches in oxygen supply and demand [26]. It is also seen in CAD corrected with PCI, and this may result in ventricular remodeling and cardiac dysfunction when present for a long time, leading to an increase in the incidence of ischemic events and mortality [27]. Deficiency anemias and coagulopathy were associated with an increased risk of in-hospital mortality by 1.6 to three times. Also, these comorbidities were prevalent in cancer patients, which further increases the risk of post-PCI mortality.

A meta-analysis study found an average of 8.1% of the patients admitted for ACS had a history of cancer. Cancer patients were older and had a higher comorbidity burden as compared to non-cancer patients, predisposing them to worse outcomes post-PCI. Also, in our study, the post-PCI inpatients with cancer were older than the non-cancer cohort. Fibrinolysis and the production of procoagulants such as tissue factor and inflammatory cytokines by the tumor, along with tumor cell-induced platelet aggregation, lead to a prothrombotic state of cancer and attributes to increased post-PCI mortality. Cancer patients also tend to receive less optimal medical therapy and have an increased risk of stent thrombosis [28]. Our study demonstrated that cancer is an independent risk factor for post-PCI mortality after controlling for demographics and other comorbidities.

A few limitations in our study include being an observational cross-sectional study, a causal relationship could not be explored between cancer, other comorbidities, and post-PCI mortality. Also, in-hospital mortality in the NIS is all-cause in post-PCI inpatients, so it is not clear whether the mortality is due to cardiovascular cause or mainly cancer deaths. Based on the administrative nature of the NIS, the database is subject to coding errors, as well as under-reporting/over-reporting of the comorbidities. Some of the strengths of our study include the utilization of the NIS data covering 44 states in the US, and our results have appropriate external validity to the American population. We used logistic regression analysis adjusted for demographic and comorbidities and evaluated the co-diagnosis of cancer risk of association with in-hospital mortality.

## Conclusions

Post-PCI patients with cancer were majorly older white men with a higher prevalence of comorbidities like coagulopathy and deficiency anemias. These cancer patients constitute a growing and high-risk population undergoing PCI. Cancer is an independent risk factor increasing the risk of in-hospital mortality by 88% in post-PCI inpatients. This association calls for an integrated care model in the management of these patient populations with cancer and other comorbidities. Collaboration between multidisciplinary cardiology and oncology teams is vital to determine the best approach to minimize mortality in cancer patients undergoing PCI.
